# Experimental Evaluation of the Rheological Properties and Influencing Factors of Gel Fracturing Fluid Mixed with CO_2_ for Shale Gas Reservoir Stimulation

**DOI:** 10.3390/gels8090527

**Published:** 2022-08-23

**Authors:** Mingwei Wang, Wen Wu, Shuyang Chen, Song Li, Tao Li, Gensheng Ni, Yu Fu, Wen Zhou

**Affiliations:** 1 School of Oil & Natural Gas Engineering, Southwest Petroleum University, Chengdu 610500, China; 2 Development Division, PetroChina Southwest Oil and Gasfield Company, Chengdu 610041, China; 3 Sinopec Northwest Oilfield Company, Urumqi 830000, China; 4 Engineering Research Institute, PetroChina Southwest Oil and Gasfield Company, Chengdu 610017, China; 5 Shunan Gas Mine, PetroChina Southwest Oil and Gasfield Company, Luzhou 646000, China; 6 State Key Laboratory of Oil and Gas Reservoir Geology and Exploitation, Chengdu University of Technology, Chengdu 610059, China

**Keywords:** shale gas reservoir, CO_2_ foam fracturing, gel fracturing fluid, foam quality, rheological property

## Abstract

Foam gel fracturing fluid has the characteristics of low formation damage, strong flowback ability, low fluid loss, high fluid efficiency, proper viscosity, and strong sand-carrying capacity, and it occupies a very important position in fracturing fluid systems. The rheological properties of gel fracturing fluid with different foam qualities of CO_2_, under different experimental temperatures and pressures, have not been thoroughly investigated, and their influence on it was studied. To simulate the performance of CO_2_ foam gel fracturing fluid under field operation conditions, the formula of the gel fracturing fluid was obtained through experimental optimization in this paper, and the experimental results show that the viscosity of gel fracturing fluid is 2.5 mPa·s (after gel breaking at a shear rate of 500 s^−1^), the residue content is 1.3 mg/L, the surface tension is 25.1 mN/m, and the interfacial tension is 1.6 mN/m. The sand-carrying fluid has no settlement in 3 h with a 40% sand ratio of 40–70-mesh quartz sand. The core damage rate of foam gel fracturing fluid is less than 19%, the shear time is 90 min at 170 s^−1^ and 90 °C, the viscosity of fracturing fluid is >50 mPa·s, and the temperature resistance and shear resistance are excellent. The gel fracturing fluid that was optimized was selected as the base fluid, which was mixed with liquid CO_2_ to form the CO_2_ foam fracturing fluid. This paper studied the rheological properties of CO_2_ foam gel fracturing fluid with different CO_2_ foam qualities under high temperature (65 °C) and high pressure (30 MPa) and two states of supercooled liquid (unfoamed) and supercritical state (foamed) through indoor pipe flow experiments. The effects of temperature, pressure, shear rate, foam quality, and other factors on the rheological properties of CO_2_ foam gel fracturing fluid were considered, and it was confirmed that among all the factors, foam quality and temperature are the main influencing factors, which is of great significance for us to better understand and evaluate the flow characteristics of CO_2_ foam gel fracturing fluid and the design of shale gas reservoir fracturing operations.

## 1. Introduction

The use of foam gel fracturing fluid is a great achievement of liquid technology. Foamed fracturing fluid is formed by dispersing N_2_ or CO_2_ in water, acid, methanol/water mixture, or hydrocarbon liquid as bubbles, and is usually a two-phase mixture of 70%–80% dryness gas (N_2_ or CO_2_) and fracturing of fluid (water-based polymer solution). Foam gel fracturing fluid is essentially a kind of gas-in-liquid emulsion, and bubbles provide high viscosity and excellent proppant-carrying capacity. Because it has the characteristics of low reservoir damage, strong flowback ability, low fluid loss, high fluid efficiency, proper viscosity, and strong sand-carrying capacity, it occupies a very important position in fracturing fluid systems.

To solve the shortcomings and defects of conventional fracturing technology, researchers began to study foam fracturing technology in the 1970s [1]. Since foam fracturing was first completed in Lincoln County, West Virginia, USA, foam fracturing technology has developed from the initial N_2_ foam fracturing to the present CO_2_ foam fracturing. In 1986, in the Federal Republic of Germany, 60% CO_2_ foam gel fracturing fluid was used in the carboniferous gas reservoir in Fez Dolf, which was buried 3400−3650 m underground. The fracturing was successful, and the natural gas production increased by nearly 12 times after fracturing [2]. By the 1990s, about 90% of gas wells and 30% of oil wells in the United States and Canada had adopted CO_2_ foam fracturing technology [3]. Nowadays, it is very common to use foam fracturing technology for fracturing worldwide. In the United States, about 3600 foam fracturing operations are carried out every year, which not only has a high success rate but also has an obvious effect on increasing production. Foams have been considered the most attractive and preferred fluid for fracturing unconventional reservoirs due to their ability to reduce formation damage and improve the recovery of injected fluid [4,5]. Gel and foam systems, as the two most widely used plugging agents for lost circulation control, have achieved positive progress in both laboratory experiments and field applications in recent decades [6,7]. Wang et al. (2022) developed a composite gel foam plugging system, which is used to plug and control flooding for heterogeneous reservoirs, and it showed better plugging and recovering performance for field applications [8].

The characteristics of CO_2_ foam fracturing fluid can also influence the propagation of hydraulic fractures. Several authors reported that the high performance of CO_2_ foam is attributed to its unique and favorable rheological characteristics [9,10,11,12,13]. However, due to the complex nature of foam, it is difficult to understand and model its flow behavior, especially under operating conditions. The versatility and uniqueness of foam are attributed to its enormously high viscosity profile compared to its base fluids and the efficiency of foam fracturing is dictated by the complex non-Newtonian behavior of foam [14,15,16,17,18]. Numerous authors agree that the design of fracturing treatments highly depends on foam rheology and it governs the overall process performance [19,20,21,22,23,24,25,26]. Foam rheology also determines the properties of the fracture network that may help in obtaining the required fracture geometry. At present, due to the limitation of equipment conditions and research methods, the research on the rheological properties of fracturing fluid under simulated field construction conditions has not been reported. The prediction of foam rheological behavior is a complex task and the direct determination of foam rheology under operating conditions is still considered a challenge [27]. Fu et al. (2021) investigated the rheology and stability of nanoparticle-stabilized CO_2_ foams under reservoir conditions (high temperature and high pressure) for fracturing applications [28]. Li et al. (2022) investigated the rheology properties of thickened liquid CO_2_ by measuring the viscosity of thickened liquid CO_2_ in different physical parameters of this prepared thickener and explained the causes of the rheological changes [29]. Kadafur et al. (2022) investigated the rheology of a CO_2_ foamed chelating agent, L-glutamic acid-N, N-diacetic acid (GLDA), which was conducted at 100 C, 1000 psi, 3.5 pH level, and various water salinities, resembling harsh reservoir conditions [30]. Tariq et al. (2022) established a data pool, which was analyzed using four machine learning techniques: Artificial Neural Network (ANN), Decision Trees (DT), Random Forest Regressor (RFR), and K-Nearest Neighbor (KNN), and it provides a simplified ANN-based model which can be used on the fly to predict the effective bulk foam viscosity in both laboratory and field conditions [31].

Presently, the limit pressure of the experimental system for studying the rheological properties of CO_2_ foam gel fracturing fluid is only 2000 psi (13.8 MPa). Under the condition of simulating tubing or formation temperature (30−50 °C), the gas phase of the foam gel fracturing fluid is in a gas state, so the foam gel fracturing fluid in the experiment is in a gas-liquid two-phase flow, while for the actual fracturing technology, the pumping pressure is extremely high, reaching tens of MPa. At the same time, the fluid temperature in the wellbore or formation fracture is also high. For CO_2_ foam gel fracturing fluid, CO_2_ is in a supercritical fluid state. The physical property of the supercritical fluid is closer to that of liquid, and the rheological property at this time is closer to that of a liquid–liquid emulsion. Most of the literature has investigated the rheological performance of the CO_2_ foam gel fracturing fluid unfoamed and ignored the different foam qualities’ effects in the foaming process, and the experimental temperature and pressure are so low that they are unable to simulate actual field fracturing conditions. Therefore, it is very important to study the rheological properties of foam gel fracturing fluid in the two states of supercooled liquid (unfoamed) and supercritical state (foamed) under simulated actual construction conditions—high pressure (tens of MPa) and high shear rate—for the effective implementation of fracturing technology, the selection of reasonable fracturing parameters, more accurate fracturing prediction, and the evaluation of fracturing effects.

To simulate the rheological performance of CO_2_ foam fracturing fluid in the two states of foamed and unfoamed under field operation conditions, the formula of the gel fracturing fluid is obtained through experimental optimization firstly, and the viscosity, static sand setting performance, and rheological performance of the foam gel fracturing fluid are experimentally evaluated. The goal is to obtain a foaming gel fracturing fluid with good performance parameters and which is mixed with liquid CO_2_. This paper selects the foam fracturing fluid formed by CO_2_ and gel fracturing fluid and studies the rheological properties of CO_2_ foam fracturing fluid under high temperature (65 °C) and high pressure (30 MPa), considering the two states of supercooled liquid and supercritical through indoor pipe flow experiments. The effects of temperature (15–90 °C), pressure (10, 20, 30 MPa), shear rate (100–3000 s^−1^), foam quality (0, 45, 55, 65, 75%), and other factors on the rheological properties of fracturing fluid are investigated, which is of great significance for better understanding and evaluating the flow characteristics of CO_2_ foam gel fracturing fluid and on-site fracturing construction design.

## 2. Results and Discussion

### 2.1. Experimental Study on the Rheological Characteristics of CO_2_ Foam Gel Fracturing Fluid

The effects of temperature, pressure, shear rate, and foam quality on the rheological properties of fracturing fluid are considered. In this experiment, the inner diameter of the test instrument pipeline was 12 mm. The effective viscosity of the CO_2_ foam gel fracturing fluid changed with the shear rate at 20 MPa, 30 MPa, and 40 MPa, and the temperature changed from 0 to 80 °C.

#### 2.1.1. Effect of Shear Rate on the Effective Viscosity of CO_2_ Foam Gel Fracturing Fluid

In actual fracturing construction, high-pressure supercooled liquid CO_2_ is often mixed with guanidine gum and then injected into the formation by tubing for fracturing. As the fracturing fluid enters the formation, the temperature gradually rises, and the high-pressure CO_2_ completely changes from supercooled liquid to a supercritical state, and the effects of CO_2_ in the two states on the effective viscosity of CO_2_ foam gel fracturing fluid are completely different. Therefore, the research on the influence of various factors on the effective viscosity of CO_2_ foam gel fracturing fluid is divided into two processes for analysis. The CO_2_ foam fracturing fluid with CO_2_ in the liquid state is defined as the fracturing fluid system under unfoamed conditions, and the CO_2_ foam fracturing fluid with CO_2_ in the supercritical state is defined as the fracturing fluid system under foamed conditions.

**Figure 1:**
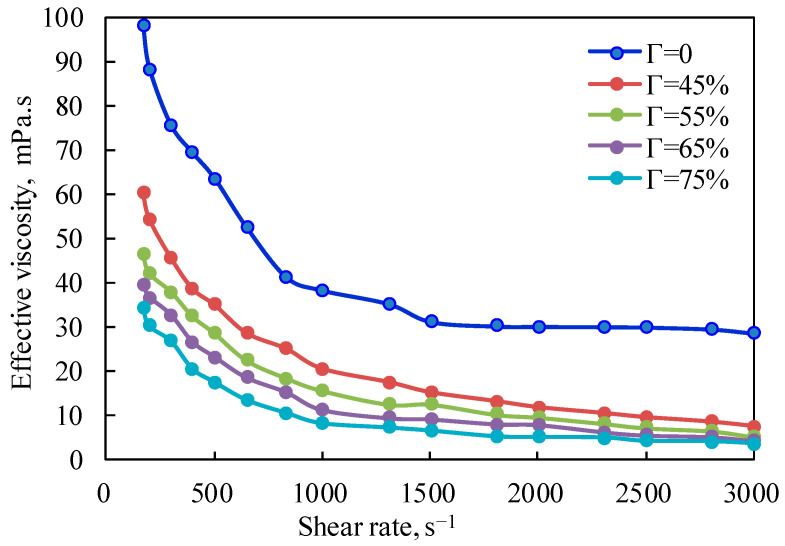
Variation curve of effective viscosity with shear rate (unfoamed, T = 20 °C).

Figure 1 is the curve of the effective viscosity of the fracturing fluid under the unfoamed condition, with a pressure of 10 MPa and a temperature of 20 °C changing with the shear rate. It can be seen from the figure that the effective viscosity of the fluid decreases exponentially with the increase in the shear rate at the same temperature, which fully shows that the unfoamed fracturing fluid is a typical shear-thinning non-Newtonian fluid, and the changing trend when the shear rate is lower than 500 s^−1^ is other than that when the shear rate is higher than 500 s^−1^. The shear-thinning characteristics of the foam system in the unfoamed state are mainly due to the influence of shear on the base liquid of the gel fracturing fluid. Linear guanidine gum is a long-chain polymer without a cross-linking structure. Increasing the shear rate will reduce the intermolecular interaction force caused by polymer molecular entanglement and hydrogen bonding, which will lead to a decrease in effective viscosity.

**Figure 2:**
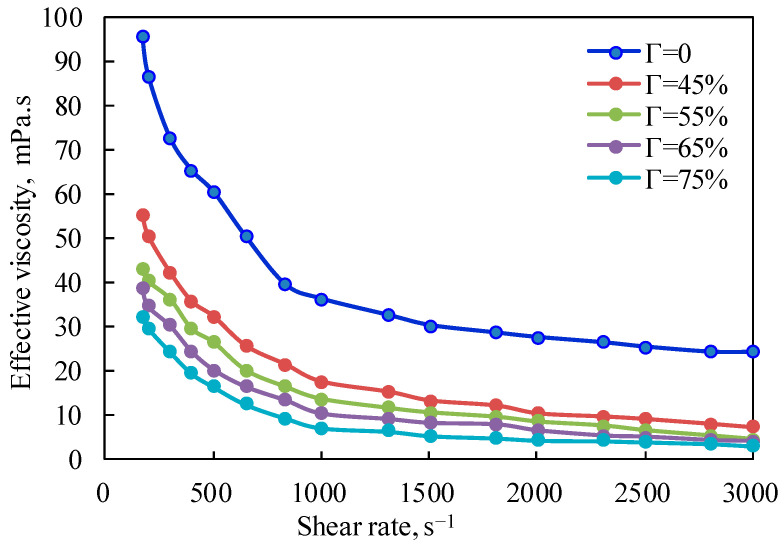
Variation curve of effective viscosity with shear rate (foamed, T = 65 °C).

Figure 2 is the curve of the effective viscosity of CO_2_ foam gel fracturing fluid with a shear rate at a pressure of 10 MPa and a temperature of 65 °C. It can be seen from the figure that the effective viscosity of CO_2_ foam gel fracturing fluid decreases exponentially with the increase in the shear rate at the same pressure, indicating that the CO_2_ foam gel fracturing fluid system is a typical shear-thinning non-Newtonian fluid, with a changing trend when the shear rate is lower than 1000 s^−1^. It can be seen from the figure that for CO_2_ foam gel fracturing fluid during foaming, CO_2_ is in a supercritical state, and its physical properties are increasingly close to those of gas. At this time, the emulsion formed by guanidine gum solution and supercritical CO_2_, which are two limited miscible fluids, is closer to the traditional foam system. The weakening effect of shearing on the viscosity of CO_2_ foam fluid is mainly reflected in two aspects: on the one hand, the shearing mentioned above will reduce the intermolecular interaction force caused by polymer molecular entanglement and hydrogen bonding; on the other hand, it is due to the destruction of the internal-phase CO_2_ foam structure by shearing.

#### 2.1.2. Effect of Foam Quality on the Effective Viscosity of CO_2_ Foam Gel Fracturing Fluid

**Figure 3:**
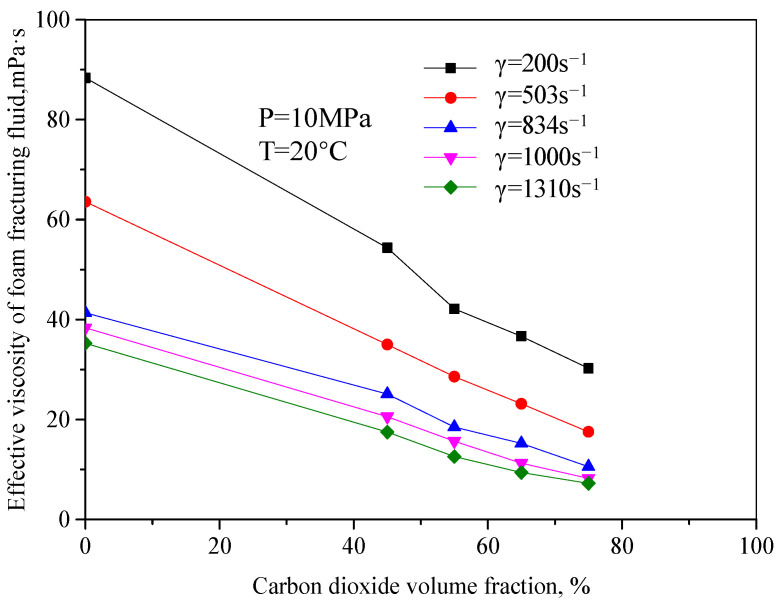
The curve of effective viscosity changes with CO_2_ volume fraction (Unfoamed, T = 20 °C).

Figure 3 is the curve of the variation of effective viscosity of fracturing fluid with a CO_2_ volume fraction when the pressure is 10 MPa and the temperature is 20 °C. It can be seen from the figure that the effective viscosity of unfoamed fracturing fluid decreases with the increase in the CO_2_ volume fraction, and the change range is large. The main reason is that the unfoamed CO_2_ is in the supercooled liquid form, similar to Newtonian fluid in this mixed system. At this time, CO_2_ has little significance for the viscosity increase in the whole system. On the contrary, the increase in the CO_2_ volume fraction will dilute the guanidine gum base liquid. When the volume shares of CO_2_ increase to a certain extent, the fluid-structure will suddenly change, from the previous guanidine gum base liquid as the continuous phase to the liquid one. When the guanidine gum base liquid changes from the external phase to the internal phase, the continuous phase of the fluid becomes the liquid CO_2_, which greatly reduces the viscosity of the whole system.

**Figure 4:**
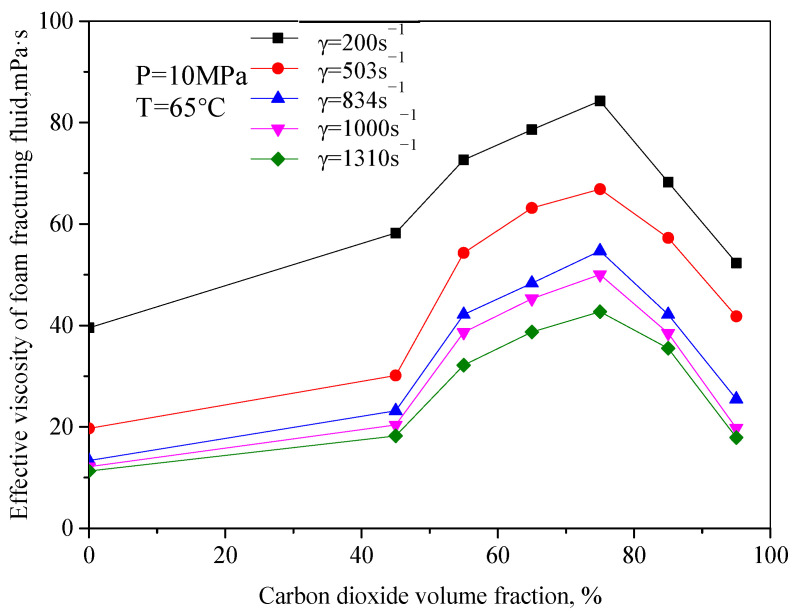
Variation curve of effective viscosity with foam quality (Foamed, T = 65 °C).

Figure 4 is the variation law curve of the effective viscosity of CO_2_ foam gel fracturing fluid with foam quality under foaming conditions, with a pressure of 10 MPa and temperature of 65 °C. It can be seen from the figure that the change rule of effective viscosity with foam quality is opposite to that without foam. The increase in foam quality makes the effective viscosity of the whole system increase, and the increased range is large. For example, when the shear rate is 834 s^−1^, the viscosity of foam gel fracturing fluid increases from 26.45 mPa·s to 56.32 mPa·s, with an increasing range of 113%. When the foam mass is 75%, the effective viscosity reaches the maximum value, and when the foam mass is more than 75%, the viscosity of foam gel fracturing fluid decreases obviously. In the research of this system, it can be seen that when the foam mass is more than 55%, with the increase in foam mass, the number of bubbles in the foam system increases, the mutual interference, and deformation among bubbles increase, the bubble structure becomes denser, and the viscosity of the foam system continues to increase.

#### 2.1.3. Effect of Temperature on Effective Viscosity of CO_2_ Foam Gel Fracturing Fluid

The effective viscosity of CO_2_ foam gel fracturing fluid with a foam mass of 45−75% was tested at elevated temperatures, and the influence of temperature on the effective viscosity of CO_2_ foam gel fracturing fluid was analyzed. In the experiment, a pipe diameter of 12 mm, a shear rate of 170 s^−1^, a heating rate of 1 °C/min, and a temperature of 80 °C were selected, and the viscosity−temperature characteristics of CO_2_ foam gel fracturing fluid under conditions of 10−40 MPa were tested.

**Figure 5:**
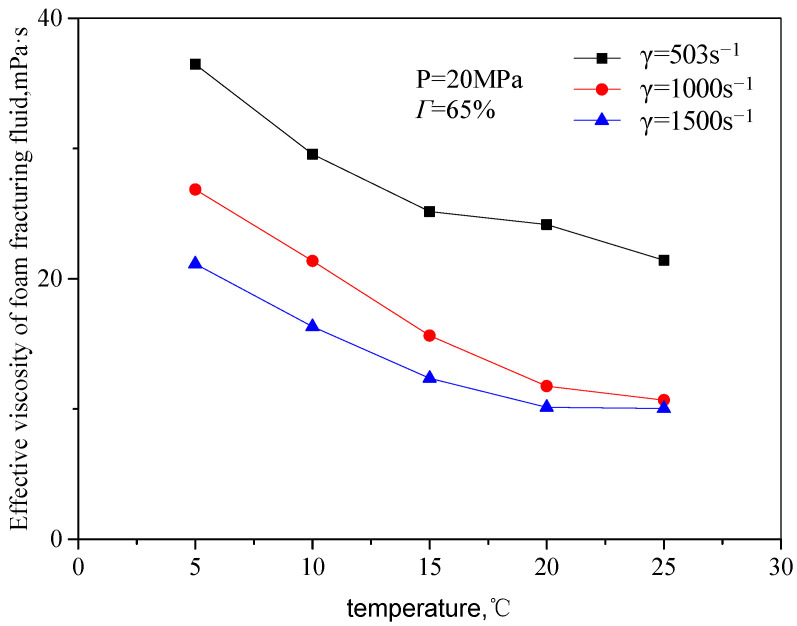
Variation curve of effective viscosity with temperature (unfoamed, 65% by volume).

**Figure 6:**
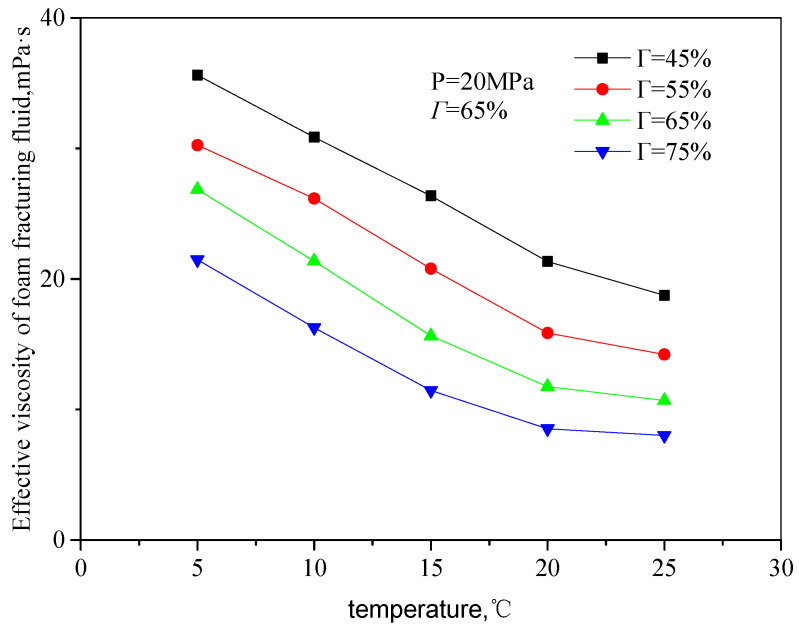
Variation curve of effective viscosity with temperature (unfoamed, shear rate 1000 s^−1^).

Figure 5 and Figure 6 are the curves of the effective viscosity of the fracturing fluid with temperature in an unfoamed state, pressure 20 MPa, and different shear rates and CO_2_ volume fractions. The temperature ranges from 5 to 25 °C, the shear rate ranges from 503 s^−1^ to 1500 s^−1^, and the volume fraction of CO_2_ from 45 to 75%. It can be seen from the figure that the effective viscosity of unfoamed fluid decreases with the increase in temperature, showing an exponentially decreasing trend. The main reason is the influence of temperature on the rheological properties of the guanidine gum base liquid: with the increase in temperature, the movement activity of guanidine gum molecules increases, and the thermal fracture of the hydrogen bonds of linear guanidine gum in the n liquid system is accelerated, so that the activation energy of guanidine gum base liquid decreases, which comprehensively shows that the effective viscosity of the solution decreases.

**Figure 7:**
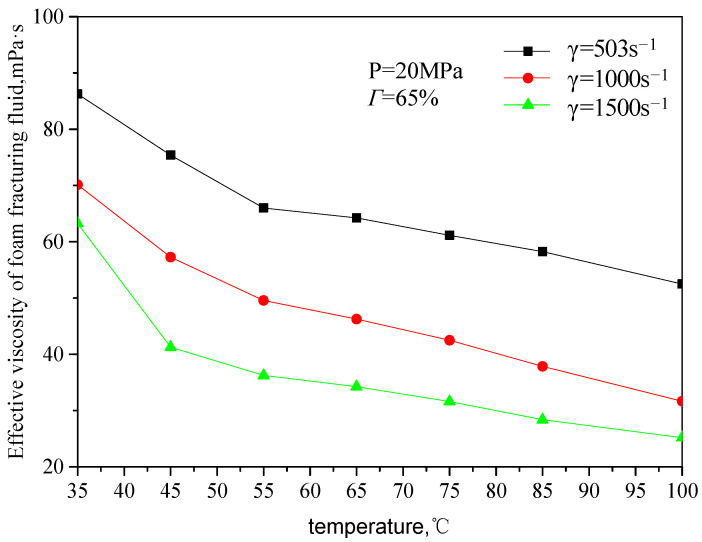
Variation curve of effective viscosity with temperature (foamed, foam mass is 65%).

**Figure 8:**
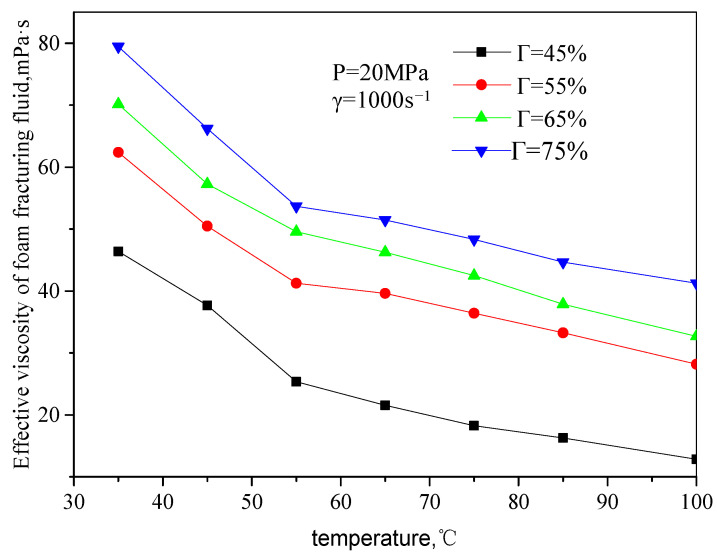
Variation curve of effective viscosity with temperature (foamed, the shear rate is 1000 s^−1^).

Figure 7 and Figure 8 are the curves of the effective viscosity of CO_2_ foam gel fracturing fluid with temperature under the foaming condition, with a pressure of 20 MPa, different shear rates, and foam quality. The temperature ranges from 35 to 75 °C, the shear rate ranges from 503 s^−1^ to 1500 s^−1^, and the foam mass ranges from 45 to 75%. It can be seen from the figure that the effective viscosity−temperature characteristics of CO_2_ foam gel fracturing fluid during foaming are consistent with those of unfoamed fracturing fluid, showing an exponentially decreasing trend. At the same time, when the temperature is greater than 55 °C, the variation ranges of the effective viscosity of foam gel fracturing fluid with temperature becomes smaller.

#### 2.1.4. Effect of Pressure on the Effective Viscosity of CO_2_ Foam Gel Fracturing Fluid (Shear Rate)

**Figure 9:**
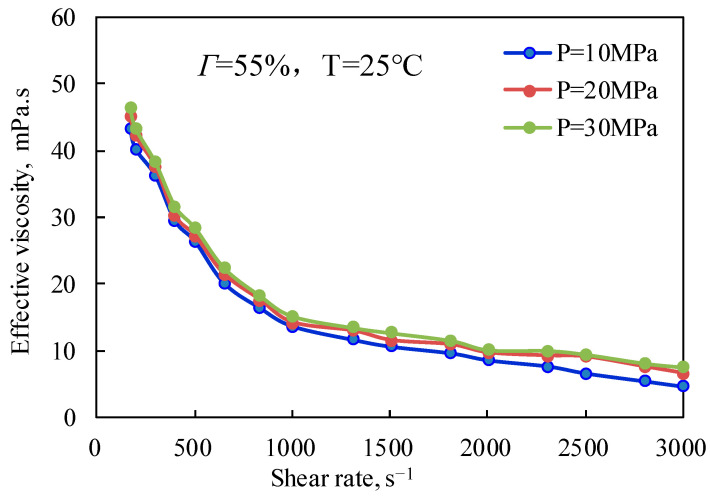
Variation of effective viscosity with shear rate under different pressures (unfoamed).

**Figure 10:**
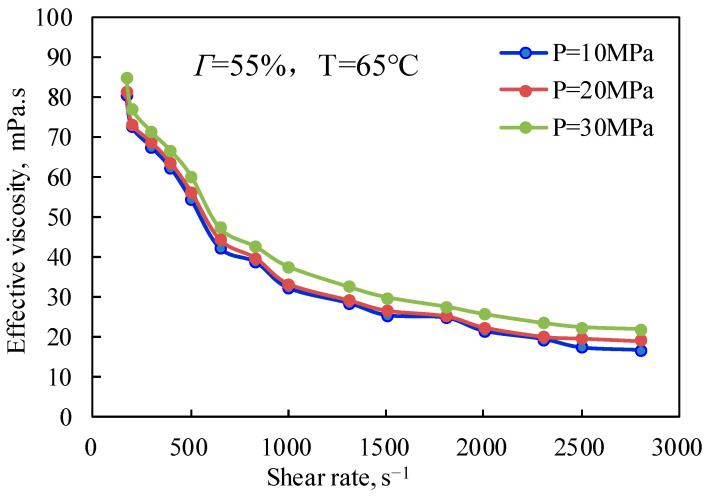
Variation of effective viscosity with shear rate under different pressures (foamed).

Figure 9 and Figure 10 are the curves of the effective viscosity of CO_2_ foam gel fracturing fluid with a shear rate under different pressure conditions and under unfoamed and foamed conditions at 25 °C and 65 °C, respectively. It can be seen from Figure 9 that the effective viscosity of the unfoamed fracturing fluid system is affected very little by pressure, and the effective viscosity increases slightly with the increase in experimental pressure. The influence of pressure on the effective viscosity of fracturing fluid without foaming is mainly that pressure can effectively change the interaction between guar gum molecules, making the linear structure change and improving the stability of the linear structure, which shows that the increase in pressure strengthens the long-chain linear structure of guar gum, slows down the damage of shear to the linear structure, and makes the viscosity of the solution increase to a certain extent. Figure 10 shows the variation law of the effective viscosity of CO_2_ foam gel fracturing fluid with pressure under the conditions of foaming at 65 °C, 55% foam mass, and 10 MPa, 20 MPa, and 30 MPa respectively. Similar to the unfoamed condition, the effective viscosity is less affected by pressure due to the pressure’s influence on bubble size and distribution in the foam gel fracturing fluid. The results show that the diameter of bubbles in the foam system gradually decreases with the increase in pressure, and the higher the pressure, the more uniform the bubble size distribution. On the one hand, the stability of bubbles is increased, while on the other hand, the nonlinear interaction between bubbles is enhanced, and the viscosity of the foam gel fracturing fluid is improved as a whole.

### 2.2. Changes in the Rheological Parameters of CO_2_ Foam Gel Fracturing Fluid with Various Factors

**Figure 11:**
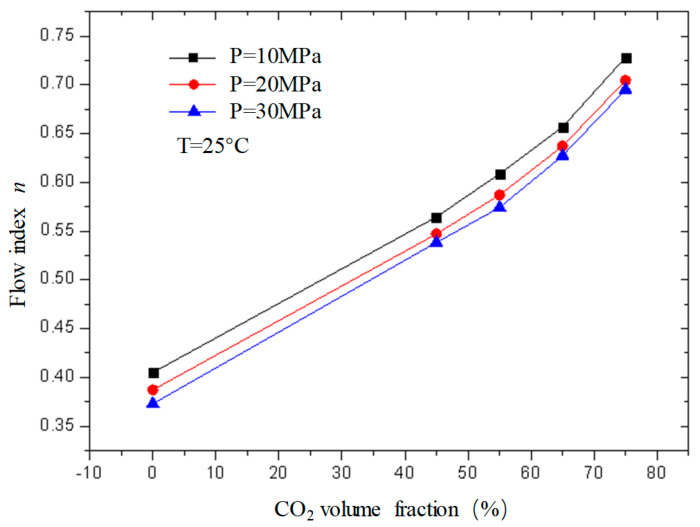
Variation curve of flow index *n* with CO_2_ volume fraction under different pressures (unfoamed).

**Figure 12:**
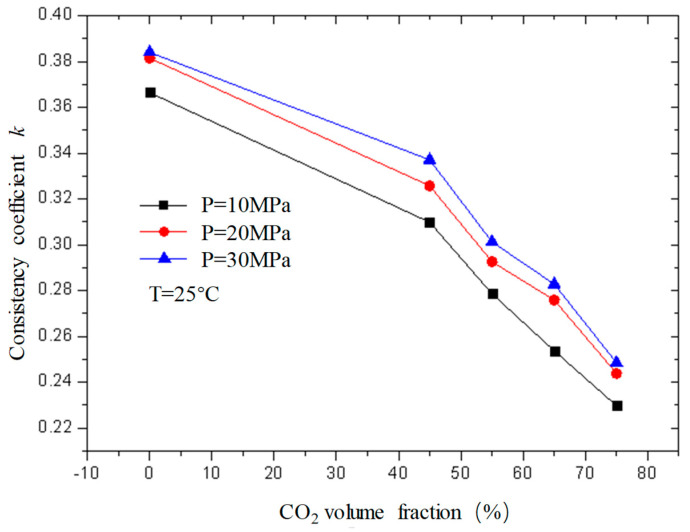
Variation curve of consistency index with CO_2_ volume fraction under different pressures (unfoamed).

Figure 11 and Figure 12 are curves of the flow index and consistency index of fracturing fluid with CO_2_ volume fraction when the temperature is 25 °C and the pressure is 10 MPa, 20 MPa, and 30 MPa, respectively. It can be seen from the figure that, when unfoamed, the flow index gradually increases with the increase in CO_2_ volume fraction, while the consistency index decreases. Under experimental conditions, the change range of the flow index is 0.37−0.72; the variation range of the consistency index is 0.23−0.39.

The flow index is a parameter used to describe the non-Newtonian property of the fluid. It can be seen from the figure that the flow index *n* of the unfoamed fracturing fluid is less than 1, indicating that the fluid is a shear-thinning non-Newtonian fluid, and the value of n is closer to 1 with the increase in CO_2_ volume fraction, indicating that the fluid property of the fracturing fluid gradually changes to a Newtonian fluid with the increase in the CO_2_ volume fraction, which is precise because the unfoamed CO_2_ exists as a supercooled liquid similar to a Newtonian fluid, and its non-Newtonian property weakens with the increase in the CO_2_ volume fraction.

#### 2.2.1. Effect of Pressure on the Effective Viscosity of CO_2_ Foam Gel Fracturing Fluid (Foam Quality)

**Figure 13:**
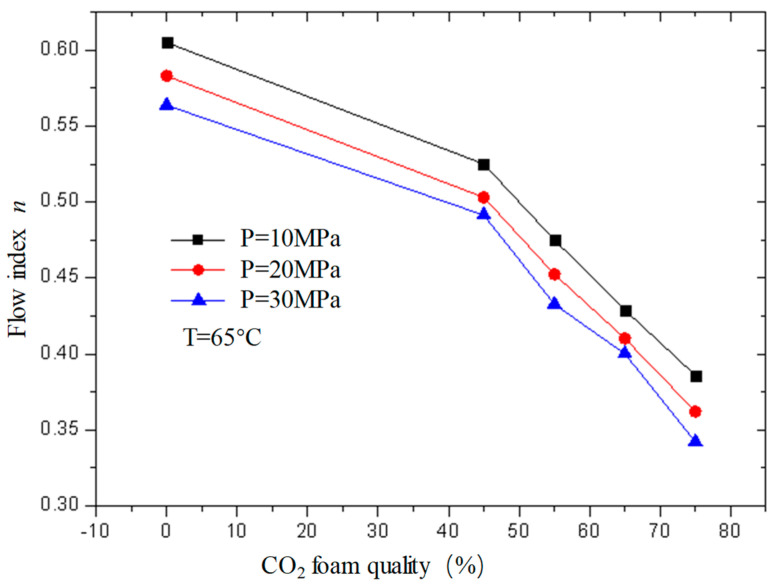
Variation curve of flow index *n* with foam quality under different pressures (foamed).

**Figure 14:**
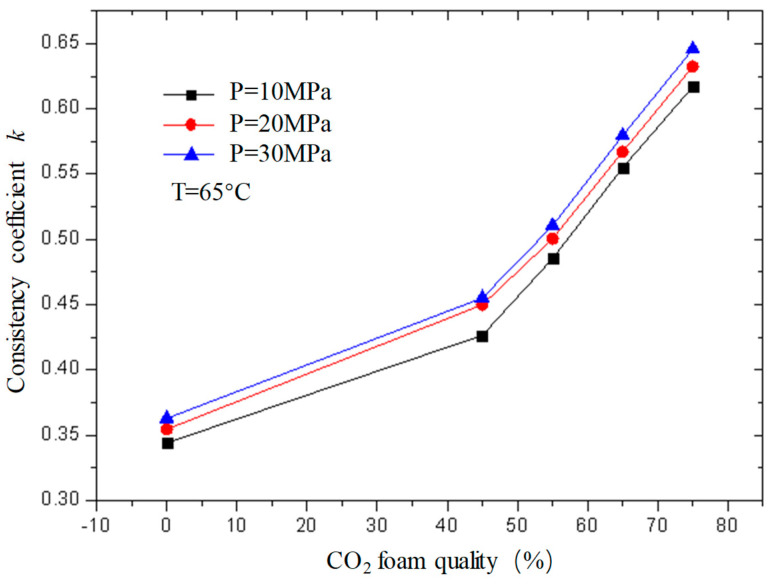
Variation curve of consistency index with foam quality under different pressures (foamed).

Figure 13 and Figure 14 are the curves of the flow index and consistency index of CO_2_ foam gel fracturing fluid with foam quality when the temperature is 65 °C and the pressure is 10 MPa, 20 MPa, and 30 MPa, respectively. As can be seen from the figure, with the increase in foam quality, the flow index gradually decreases, and the consistency index increases, and from the perspective of the change range, it starts to increase substantially when the foam quality is 55%. This shows that the CO_2_ foam gel fracturing fluid in the foaming stage still belongs to the shear-thinning non-Newtonian fluid, and with the increase in foam quality, the non-Newtonian fluid properties of the foam gel fracturing fluid gradually increase.

#### 2.2.2. Effect of Temperature on the Rheological Parameters of CO_2_ Foam Gel Fracturing Fluid

**Figure 15:**
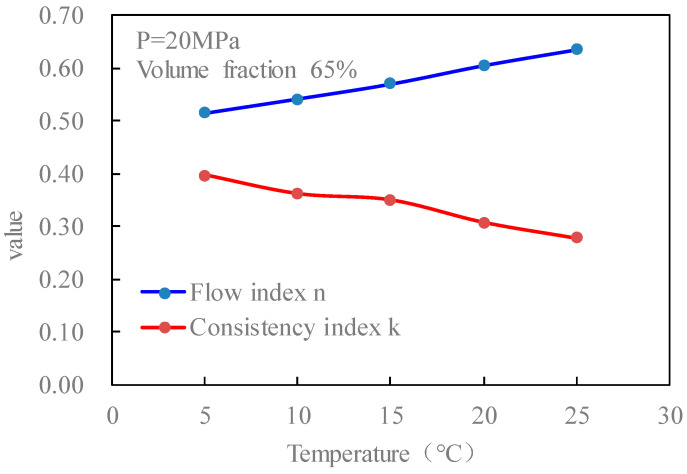
Variation curves of flow index *n* and consistency index k with temperature (unfoamed).

Figure 15 is the curves of the flow index and consistency index of fracturing fluid with temperature when the pressure is 20 MPa and the volume fraction of CO_2_ is 65% without foaming. It can be seen from the figure that with the increase in temperature, the flow index of the fluid increases, and the consistency index decreases.

**Figure 16:**
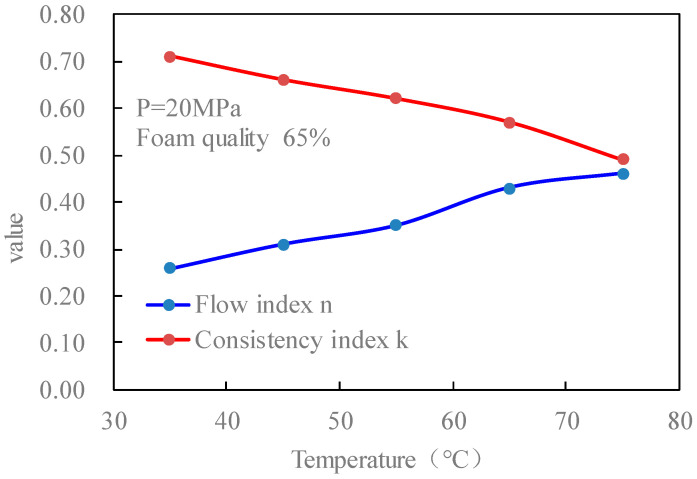
Variation curves of flow index *n* and consistency index *k* with temperature (foamed).

Figure 16 is the curves of the flow index and consistency index of the CO_2_ foam gel fracturing fluid with temperature when the pressure is 20 MPa and the foam mass is 65%. It can be seen that the change rule of rheological parameters of foamed fracturing fluid with temperature is consistent with that of unfoamed fracturing fluid, which shows that with the increase in temperature, the flow index of fluid increases and the consistency index decreases. From the action mechanism, it is the same as that of the previous temperature on the viscosity characteristics of CO_2_ foamed fracturing fluid.

#### 2.2.3. Effect of Pressure on the Rheological Parameters of CO_2_ Foam Gel Fracturing Fluid

**Figure 17:**
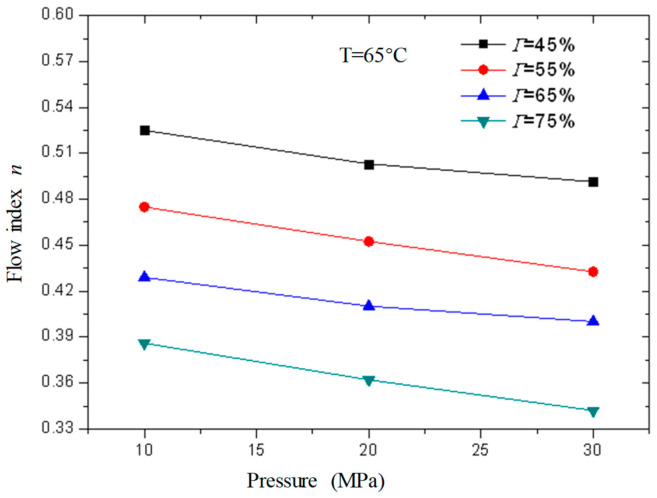
Variation curve of flow index *n* of foam gel fracturing fluid with pressure under foaming.

**Figure 18:**
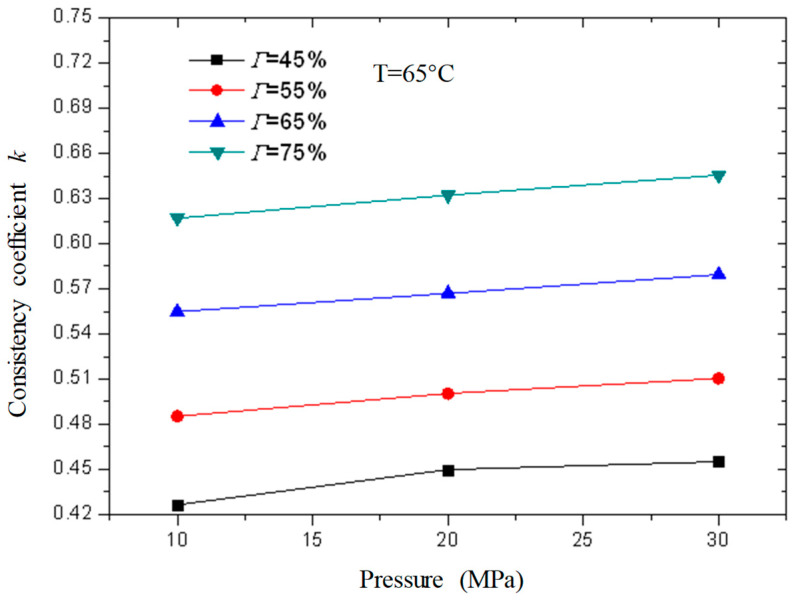
Variation curve of consistency index *k* of foam gel fracturing fluid with pressure under foaming.

Figure 17 and Figure 18 are curves of the rheological parameters of CO_2_ foam gel fracturing fluid changing with pressure when the temperature is 65 °C and the foam quality is 45%, 55%, 65%, and 75% respectively. It can be seen from the figures that with the increase in pressure, the change of fluid flow index and consistency index is very small, which shows that the influence of pressure on the rheological properties of the foam gel fracturing fluid is almost negligible compared with temperature and foam quality. Meanwhile, the influence of pressure on the rheological properties of the foam gel fracturing fluid is negligible.

### 2.3. Sensitivity Analysis

Sensitivity analysis is a method to analyze system stability in system analysis. There is a system, and its system characteristics, *P*, are mainly determined by *n* factors *a* = {*a*_1_, *a*_2_, …, *a*_n_} and *P* = *f* (*a*_1_, *a*_2_, …, *a*_n_). In a certain reference state, a* = {*a*_1_*, *a*_2_*, …, *a*_n_*}, the system characteristic is P*. Let each factor change within its possible range, and analyze the trend and degree of the deviation of the system characteristic *P* from the benchmark state P* due to these factors. This analysis method is called sensitivity analysis.

We define the dimensionless sensitivity function and sensitivity factor. That is, the ratio of the relative error of the system characteristic *P* to the relative error of the parameter *a_k_* is defined as the sensitivity function *S_k_*(*a_k_*) of the parameter *a_k_*.

(1) $$\frac{S_{k}\left(a_{k}\right)\left(\frac{\left|\Delta P\right|}{P}\right)}{\left(\frac{\left|\Delta a_{k}\right|}{a_{k}}\right)}=\left|\frac{\Delta P}{\Delta a_{k}}\right|\frac{a_{k}}{a_{k}}\;k=1,2,\ldots \ldots ,n$$

When $\left|\Delta a_{k}\right|/a_{k}$ is small, *S_k_*(*a_k_*) can be approximately expressed as:

(2) $$S_{k}\left(a_{k}\right)=\left|\frac{d\varphi_{k}a_{k}}{da_{k}}\right|\frac{a_{k}}{P}\;\;k=1,2,\ldots \ldots ,n$$

S_k_*, *k* = 1, 2, …, *n*, is a set of dimensionless non-negative real numbers. The larger the S_k_* value, the more sensitive *P* is to *a_k_* in the reference state. Through the comparison of S_k_*, the sensitivity of system characteristics to various factors can be compared and evaluated.

To understand the sensitivity of the rheological properties of foam gel fracturing fluid to various factors, the above sensitivity analysis method was used to analyze the main factors affecting the rheological properties of foam gel fracturing fluid, and the sensitivity of each factor was compared. The characteristics of the system, that is, the rheological characteristics of foam gel fracturing fluid, are characterized by the effective viscosity of the system. The parameters for sensitivity analysis are shear rate, pressure, foam quality, and temperature. Observe the shape of the rheological curve and establish the functional relationship between effective viscosity and shear rate, temperature, and other parameters to obtain the sensitivity function, and then calculate the sensitivity factor. After analysis, the sensitivity values of each parameter are obtained (Table 1), which are ranked by size, followed by foam quality, temperature, shear rate, and pressure. It can be seen that among all the factors, foam quality and temperature are the main influencing factors. Therefore, the performance of CO_2_ foam gel fracturing can be mainly regulated by the two parameters, which is helpful for application in shale gas reservoir fracturing.

## 3. Conclusions

The formula of the gel fracturing fluid was obtained through experimental optimization, which evaluated experimentally the viscosity, residue, surface tension, sand-carrying capacity, and interfacial tension of the gel fracturing fluid. The core damage rate of the gel fracturing fluid is less than 19%, the shear time is 90 min at 170 s^−1^ and 90 °C, and the viscosity of the fracturing fluid is >50 mPa·s.

The rheological properties of CO_2_ foam gel fracturing fluid and its influencing factors were studied experimentally. For the foam gel fracturing fluid with CO_2_ in supercooled liquid and at a supercritical state, the effects of temperature, pressure, shear rate, and foam quality on the rheological properties of the gel fracturing fluid were considered. The main conclusions are as follows:

In the unfoamed state, the effective viscosity of foam gel fluid decreases exponentially with the increase in shear rate, gradually decreases with the increase in CO_2_ volume fraction, and the effective viscosity of fluid decreases with the increase in temperature. The effective viscosity is little affected by pressure. In the foaming state, the change rule of effective viscosity with shear rate is the same as that without foaming, the change rule with foam quality is opposite to that without foaming, and the effective viscosity−temperature characteristics of fluid are the same as that without foaming.

Without foaming, the foam quality increases, the flow index gradually increases, and the consistency index decreases. With the increase in temperature, the flow index of fluid increases, and the consistency index decreases. When foaming, the foam quality increases, the flow index gradually decreases, and the consistency index increases. When the foam quality is about 50%, a sudden change begins. With the increase in temperature, the flow index of fluid increases, and the consistency index decreases. With the increase in pressure, the flow index and consistency index of fluid change very little.

Based on the sensitivity analysis method, the influencing factors of the rheological behavior of CO_2_ foam gel fracturing fluid are foam quality, temperature, shear rate, and pressure, in turn, which provides a theoretical basis for CO_2_ foam fracturing technology.

## 4. Experiments and Methods

### 4.1. Experimental Optimization of Gel Fracturing Fluid

#### 4.1.1. Formula Design

In recent years, the CO_2_ foam gel fracturing fluid system, which has been applied successfully to reservoir stimulations, is a cross-linked fracturing fluid system with guanidine gum as the thickener.

The commonly used foam sealing and channeling systems are mainly composed of a foaming agent, foam stabilizer, and gas phase [32,33]. Therefore, guanidine gum fluid is used as the basic liquid phase in this study, the thickener and foaming agent are optimized by experiments, and the gel fracturing fluid that is needed to mix with liquid CO_2_ in this research is formed. According to the characteristics of foam gel fracturing fluid, the high-speed mixing method (Waring Blender method) was selected to evaluate the foaming ability and foam stability of the foaming agent.

##### Single Agent Optimization

(1) Thickener optimization

To select a liquid that can form foam with good stability at a low dosage, the frequently used thickeners were evaluated and optimized by experiments, including CT5-7, CT5-7WⅠ, hydroxypropyl guar gum (CMHPG), instant carboxymethyl cellulose (CMC), and polyanionic cellulose (PAC), and the results of the thickeners’ foam stability are shown in Table 2. The experimental results showed that the foam stabilizing performance of CT5-7 is much better than the others. Therefore, the CT5-7 thickener is selected as the thickener of the gelled fracturing fluid formula.

The CT5-7 is a thickener, which is prepared from acrylamide, vinylpyrrolidone, maleic acid, anionic functional monomer, initiator, inorganic salt, etc. It includes acrylamide 25~30%, vinylpyrrolidone 3~5%, maleic acid 4~6%, anionic functional monomer 5~7%, initiator 0.08~0.15%, potassium hydroxide 1~8%, inorganic salt 5~10%, and the rest is water.

(2) Foaming agent optimization

The foaming ability and foam half-life of 0.3% CT5-7B, CT5-7S, sodium dodecylbenzene sulfonate (ABS), alkyl betaine (DSB), CT5-7C and other foaming agents in CT5-7 thickener solution were experimentally evaluated. These are the foaming agent, and their main component is alcohols; CT5-7B is fusel, CT5-7S is methyl isobutyl methanol, and CT5-7C is triethoxybutane. The results are shown in Table 3. The calculation formula of foam quality *Γ* is:

*Γ* = (V_0_ − 100)/V_0_ (3)

where V_0_ is the volume of gel fracturing fluid, mL.

The foaming power and foam stability data of five foaming agents were analyzed, and CT5-7S with good foaming power and foam stability was selected as the foaming agent of the foam gel fracturing fluid formula.

##### Optimization of a Single Dosage

(1) Thickener dosage

To optimize the amount of thickener in gel fracturing fluid, an experimental evaluation was carried out on the sand suspension (settling velocity of 40–70 mesh quartz sand) of fracturing fluid with 0.2, 0.3, 0.4, and 0.5% of thickener at 30~90 °C, and the experimental results are shown in Table 4.

The experimental results show that the sedimentation rate of quartz sand in the gel fracturing fluid increases with the increase in temperature. When the amount of CT5-7 is 0.2% and 90 °C, the suspension capacity of quartz sand is poor. When the amount is 0.3–0.4%, the sedimentation rate of quartz sand is 1–10 mm/s at 90 °C, but when the amount is increased to 0.5%, the quartz sand hardly sinks.

The analysis shows that after the gel fracturing fluid forms foam, its apparent viscosity increases, and the ability to suspend and carry solid particles increases significantly.

(2) Stability of foam with different thickener dosage

The stability (half-life) of gel fracturing fluid with thickener dosages of 0.2, 0.3, 0.4%, and 0.5% was experimentally evaluated at room temperature and up to 90 °C The dosage of 0.3% CT5-7S foaming agent was 0.3%, as shown in Table 5. It can be seen from Table 5 that with the increase in temperature, the half-life of foam decreases. At a dosage of 0.2%, the half-life is 0.2 h at 90 °C; at a dosage of 0.3~0.4%, the half-life of foam is 1.4~2.5 h at 90 °C; and at a dosage of 0.5%, the half-life of foam is 4.2 h at 90 °C.

Based on the experimental data on viscosity, sand suspension, and the half-life of foam in gel fracturing fluid, the thickener dosage of gel fracturing fluid with a foam formula is determined to be 0.5%.

(3) Determination of Foaming agent dosage

To determine the dosage of the foaming agent, the half-life of foam when the dosage of CT5-7S foaming agent is 0.1, 0.3, and 0.5%, the experimental temperature is 30 °C, and the experimental data are shown in Table 6. It can be seen from Table 6 that as the dosage of CT5-7S foaming agent increases from 0.1% to 0.5%, for CT5-7 thickener, the half-life of foam shows an increasing trend. Considering the performance and cost factors, the dosage of the CT5-7S foaming agent is determined to be 0.3%.

##### The formula of Gel Fracturing Fluid with Foam

Through the experimental evaluation of the single agent of gel fracturing fluid with foam such as foaming agent and thickener, the experimental evaluation data analysis of foam half-life, foam quality, and the viscosity of gel fracturing fluid is carried out, and the formula of gel fracturing fluid is determined: 0.5% CT5-7 thickener + 0.3% CT5-7S foaming agent + 0.3% CT5-7D high-temperature stabilizer + 0.3% CT5-7U regulator.

#### 4.1.2. Performance Testing

##### Gel Breaking Performance

The gel fracturing fluid volume of 1000 mL was prepared in the mixer, and then the 600 ppm gel breaker (ammonium persulfate) was added, which may break the gel fracturing fluid at 90 °C. The gel fracturing fluid after the gel was broken was centrifuged and dried with the separated residue at 105 °C ± 1 °C. Then, the residue content in the gel fracturing fluid was determined. The results are shown in Table 7.

The residue content in the gel breaker is calculated according to the following formula:

(4) $$\eta =\frac{m}{V}\times 1000$$

where η is the residue content in the gel breaker, mg/L, m is the residue mass in mg, and V is the volume of gel fracturing fluid in mL.

##### Sand Setting Performance at the Static Station

**Figure 19:**
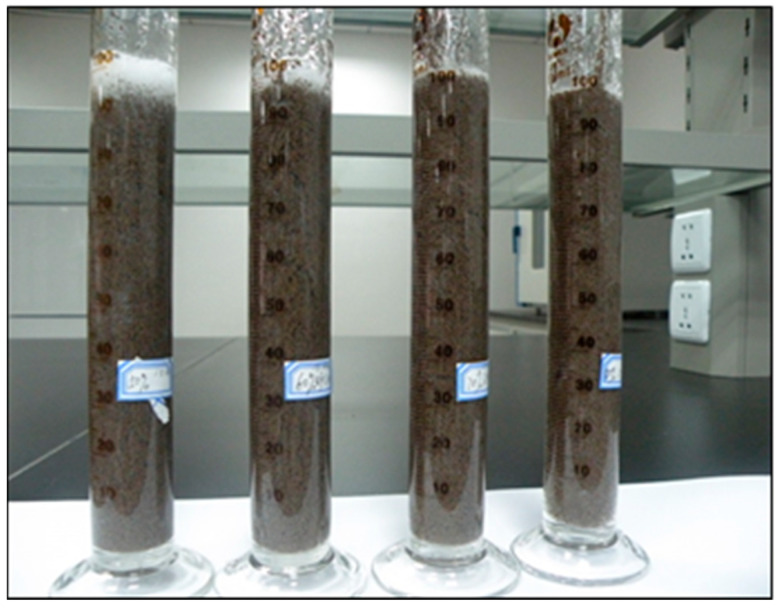
The static suspended sand test results of gel fracturing fluid.

It is necessary to test the sand carrying performance of gel fracturing fluid by a static suspended sand experiment. The gel fracturing fluid with foam quality (Q = 65%) was prepared according to the liquid formula and 200 mL of fluid was poured into the beaker and then placed in a 90 °C water bath at a constant temperature for 20 min. Then, the liquid was poured into the mixer, and 40−70 mesh quartz sand was added according to the 40% sand ratio and stirred evenly. Then, the mixed liquid with sand was poured into a 250 mL measuring cylinder and put into an oven at 90 °C (formation temperature is usually 85~100 °C). The volume of clear liquid separated from the upper layer was recorded at regular intervals. The static suspended sand test results of the gel fracturing fluid are shown in Figure 19. The experiment shows that after the gel fracturing fluid forms a stable foam, the proppant is evenly dispersed in the foam fluid. Due to the interface effect between foam, the proppant has a wrapping and supporting effect. From room temperature up to 90 °C, the sand mixing fluid remains uniform for 3 h, there is no obvious stratification phenomenon, and the sand carrying performance is good.

##### Formation Damage Evaluation

The core of the shale reservoir is used to evaluate the damage performance of gel fracturing fluid. The test results are shown in Table 8. It can be seen that the damage rate of foam gel fracturing fluid to shale is less than 19%.

##### Rheological Properties

Evaluation method: take 70 mL of gel fracturing fluid, add regulator according to the proportion of 0.3% (*v*/*v*), adjust the pH value of the liquid to 5.8, then add foaming agent according to the design proportion, mix it evenly, transfer it to the closed system of the RS6000 high-temperature rheometer, connect the CO_2_ gas source to pressurize 10 bar and ensure that the gel fracturing fluid is in the CO_2_-saturated state, and test the temperature resistance and shear resistance of the fracturing fluid. The experimental results show that the fracturing fluid can still maintain a high apparent viscosity after a long time of shearing at 90 °C at a shear rate of 170 s^−1^ and the apparent viscosity is greater than 50 mPa·s, indicating that it has excellent temperature and shear resistance.

**Figure 20:**
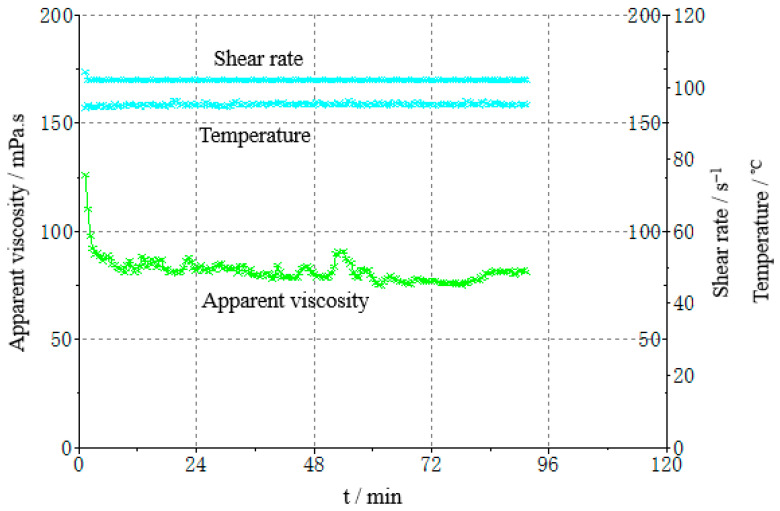
Evaluation of rheological properties of gel fracturing fluid with foam.

The formula of the gel fracturing fluid is obtained through experimental optimization, and the viscosity, static sand setting performance, and rheological performance of the foam gel fracturing fluid are experimentally evaluated. The experimental results show that the viscosity of the fracturing fluid is 2.5 mPa·s (after gel breaking and at a shear rate of 500 s^−1^), the residue content is 1.3 mg/L, the surface tension is 25.1 mN/m, and the interfacial tension is 1.6 mN/m. The 40−70 mesh quartz sand commonly used in shale gas fracturing is used for static sand setting experiments and the sand ratio is 40%. The static sand carrying experiment was carried out. The sand-carrying fluid had good flow performance, and the proppant showed no settlement in 3 h. The core of the shale reservoir was selected for the damage evaluation experiment of the foam gel fracturing fluid. The test results are shown in Figure 20. It was observed that the core damage rate of foam gel fracturing fluid is less than 19%, the shear time is 90 min at 170 s^−1^ and 90 °C, and the viscosity of the fracturing fluid is >50 mPa·s, and the temperature resistance and shear resistance are excellent.

### 4.2. Preparation Methods of Gel Fracturing Fluid with Different CO_2_ Foam Mass

**Figure 21:**
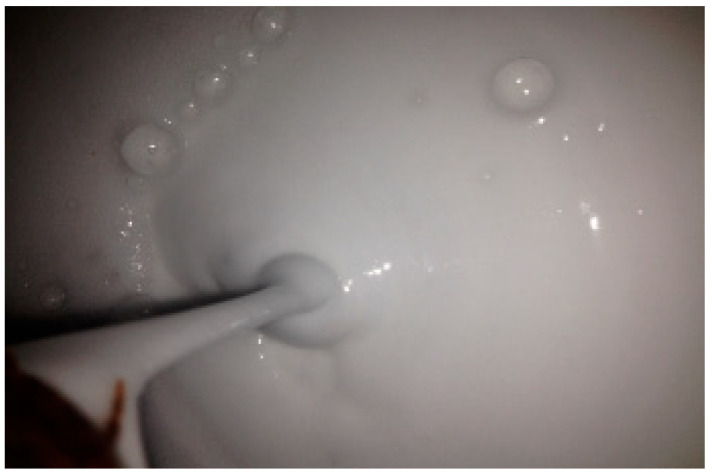
CO_2_ foam gel fracturing fluid.

According to the formula of the gel fracturing fluid optimized in Section 2.1, the gel fracturing fluid was obtained in the laboratory and stirred well to obtain the liquid-phase CO_2_ foam gel fracturing fluid (Figure 21).

The proportion of gas phase in foam gel fracturing fluid is usually described by foam quality or foam dryness, which indicates the phase’s percentage in the total volume of the foam gel fracturing fluid; that is, the gas volume contained in a unit volume of foam gel fracturing fluid.

Usually expressed by *Γ*:

(5) $$\Gamma =\frac{V_{G}}{V_{G}+V_{L}}\times 100\%=\frac{V_{G}}{V_{F}}\times 100\%$$

where: *Γ* is the foam quality; *V*_G_ is the gas volume, m^3^; *V*_L_ is the liquid volume, m^3^; and *V*_F_ is the total volume of the foam, m^3^.

Because CO_2_ is in a supercooled liquid state and supercritical state in this experiment, the foam quality is defined as the percentage of CO_2_ volume in the whole foam volume under certain temperature and pressure conditions.

### 4.3. Experimental Principle of the Rheological Properties of CO_2_ Foam Gel Fracturing Fluid

For CO_2_ foam gel fracturing fluids, within the range of practical shear rate, they are close to power-law fluids, and a set of *n* and *k* values can be used to characterize their rheological characteristics.

A thin-tube rheometer calculates the relationship between shear stress and shear rate through the measured pressure drop and flow rate of fluid in the tube, to determine the rheological characteristics of the fluid. The flow of fluid in a narrow tube better meets the following conditions: ① viscous laminar flow; ② constant flow; ③ uniform flow; ④ no-slip pipe wall.

The basic formula for the laminar flow of viscous fluid in a circular tube is:

(6) $$\bar{U}=\frac{D}{2\tau_{w}^{3}}\int_{0}^{\tau_{w}}f\left(\tau \right)\tau^{2}d\tau$$

where $\bar{U}$ is the average velocity of the fluid in the pipe, *D* is the diameter of the pipe, and $\tau_{w}$ is the wall shear stress.

Transforming Formula (6) into Formula (7):

(7) $$\frac{1}{4}\frac{8\bar{U}}{D}\tau_{w}^{3}=\int_{0}^{\tau_{w}}f\left(\tau \right)\tau^{2}d\tau$$

Formula (7) is the basic formula for the rheometer.

For power-law fluid, the constitutive formula is:

(8) $$f\left(\tau \right)={\left(\frac{\tau }{k}\right)}^{\frac{1}{n}}$$

Substituting Formula (8) into Formula (7) of tube rheometer to obtain Formula (9):

(9) $$\frac{1}{4}\frac{8\bar{U}}{D}\tau_{w}^{3}={\int_{0}^{\tau_{w}}\left(\frac{\tau }{k}\right)}^{\frac{1}{n}}\tau^{2}d\tau$$

Integrate the above formula to obtain Formula (10):

(10) $$\tau_{w}=k{\left(\frac{8\bar{U}}{D}\right)}^{n}{\left(\frac{3n+1}{4n}\right)}^{n}$$

Take the logarithm on both sides of the formula and obtain Formula (11):

(11) $$\mathrm{lg}\tau_{w}=\mathrm{lg}k{\left(\frac{3n+1}{4n}\right)}^{n}+n\mathrm{lg}\left(\frac{8\bar{U}}{D}\right)$$

Type, the wall shear stress $\tau_{w}$ is:

(12) $$\tau_{w}=\frac{\Delta pD}{4L}$$

**Figure 22:**
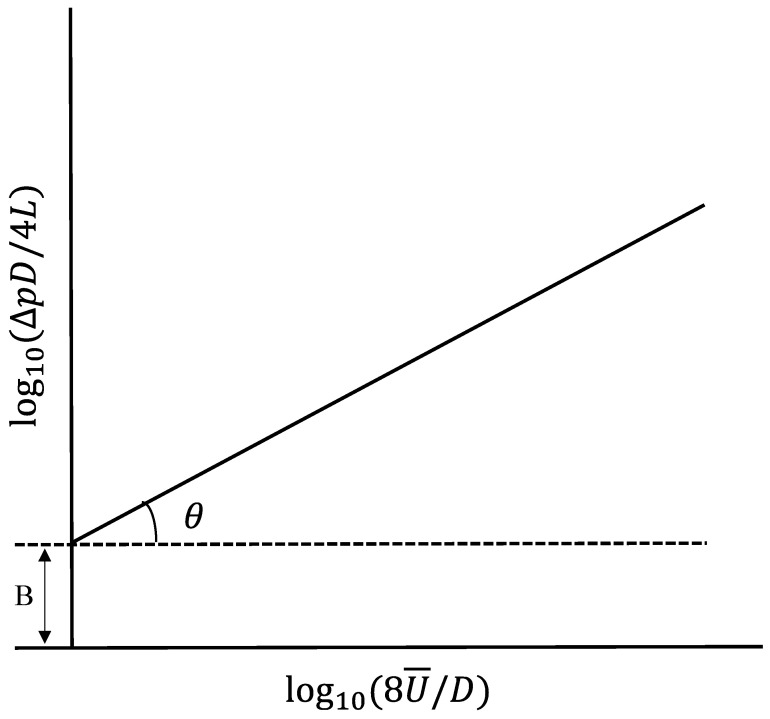
Power-law fluid flow curve (logarithmic coordinates). (Reprinted/adapted with permission from Ref. [9], 2014, copyright X. Sun et al.).

After the pressure drop and flow, *Q* is measured, and the relationship curve $\mathrm{lg}\left(\Delta pD/4L\right)\sim \mathrm{lg}\left(8\bar{U}/D\right)$ is compiled, as shown in Figure 22. From the slope tgθ and intercept *B* of the straight line, the rheological characteristic parameters *n* and *k* of the power-law fluid can be determined.

(13) n=tgθ

(14) $$k=B/{\left(\frac{3n+1}{4n}\right)}^{n}$$

## Figures and Tables

**Table 1:** Sensitivity factors of influencing factors.

Influencing Factors	Shear Rate	Pressure	Foam Quality	Temperature
Sensitivity factor	0.685	0.092	0.973	0.735
Sensitivity grade	Ⅲ	Ⅳ	Ⅰ	Ⅱ

**Table 2:** Experimental results of thickening agent foam stability.

Thickening Agent	Modulators	Foaming Agent	V_0_/mL	Foam Quality/%	The Half-Life of Foam/h
0.3% CT5-7	0.3% CT5-7U	0.3% CT5-7B	280	70.4	90
0.5% CT5-7 WⅠ			280	68.3	75
0.5% CMHPG			280	61.4	65
0.5% instant CMC			280	60.6	18
0.5% CMC			280	60.2	28
0.5% PAC			280	60.3	16

**Table 3:** Evaluation of foaming and foam stabilizing properties of the foaming agent.

Thickener	Foaming Agent	V_0_/mL	Foam Quality/%	The Half-Life of the Foam/h
0.5% CT5-7	0.3% CT5-7B	270	60.6	110
	0.3% CT5-7S	270	64.9	120
	0.3% DSB	270	64.3	95
	0.3% CT5-7C	270	64.2	100
	0.3% ABS	270	63.7	96

**Table 4:** Experimental results of gel fracturing fluid-suspended sand at different temperatures (mm/s).

CT5-7 Dosage/%	15 °C (Room Temperature)	30 °C	50 °C	70 °C	90 °C
0.2	12.5	25.0	33.3	50.0	100.0
0.3	0.70	1.29	2.32	5.56	9.52
0.4	0.019	0.057	0.11	0.70	0.98
0.5	Almost motionless	Almost motionless	0.046	0.16	0.46

**Table 5:** The half-life of foam in gel fracturing fluid at different temperatures.

CT5-7/%	15 °C (Room Temperature)		30 °C		50 °C		70 °C		90 °C	
	Foam Quality/%	Half-Life/h	Foam Quality/%	Half-Life/h	Foam Quality/%	Half-Life/h	Foam Quality/%	Half-Life/h	Foam Quality/%	Half-Life/h
0.2	75.21	89	79.13	75	79.62	40	82.32	21	93.13	0.2
0.3	71.60	92	76.27	76	76.76	60	80.53	37	92.25	1.4
0.4	69.57	109	71.43	105	74.21	70	79.16	56	91.01	2.5
0.5	66.04	133	67.03	119	71.17	90	77.45	68	89.32	4.2

**Table 6:** The half-life of foam in gel fracturing fluid with different dosages of CT5-7S.

Foaming Agent	Thickening Agent	V_0_/mL	Foam Quality/%	The Half-Life of Foam/h
0.1% CT5-7S	0.5% CT5-7	288	63.5	90
0.3% CT5-7S		292	65.7	110
0.5% CT5-7S		292	65.8	113

**Table 7:** Gel breaking performance of gel fracturing fluid.

Breaker Viscosity /mPa·s	Residue Content/mg·L^−1^	Surface Tension/mN/m	Surface Tension/mN/m
2.5	1.3	1.6	25.1

**Table 8:** Core damage performance of gel fracturing fluid.

Core No.	Gas phase Permeability before Fracturing Fluid Injection/10^−3^ μm^2^	Gas Phase Permeability after Fracturing Fluid Injection/10^−3^ μm^2^	Damage Rate/%	Average Value/%
1#	0.12863	0.10821	15.87	15.05
2#	0.09756	0.08602	11.83	
3#	0.03584	0.03023	15.65	
4#	0.02424	0.02016	16.83	

## Data Availability

Not applicable.
